# Supplementary material for: Study of thin layer film evolution near bacterial cellulose membrane by Ag|AgCl electrodes in chamber with lower concentration
Source: PLoS One. 2022 Feb 2;17(2):e0263059. doi: 10.1371/journal.pone.0263059 (PMC8809578; doi:10.1371/journal.pone.0263059)
Supplement: Attachment — Submitted filename: Response to Reviewers.docx [file pone.0263059.s002.docx]

Thank you for the valuable comments of the reviewers, which allowed us to see new research opportunities, but also identify some the shortcomings in the article, which we will try to eliminate and present certain issues more clearly, avoiding mental shortcuts that may hinder a quick analysis of the content of the article. In addition, the text was checked and improved. Significant and new changes of the text of the article are marked in yellow.

We checked and completed the << Reference list >> and tried to respond to reviewers' comments and include them in our article.

**Ad rev. 1**

1. ***Provide photos of reaction setup taken during analysis.***

We use a simplified diagram of the measuring system in the article. The measuring systems are of our own design and we paid less attention to the aesthetics of the system, while our main attention was focused on repeatability and measurement accuracy. For this reason, we believe that the simplified diagram of the system is better suited to the presentation of the main measurement idea (the simplified diagram of the system can be extended with subsystems such as: the solution mixing system (1), a millivoltmeter with computer connection (2), a system of vessels allowing us to determine the pressure in the chambers (3) - the pressure difference on the membrane, which were described in our previous publications quoted in the article).

The example photo of the measuring system is presented below.

We propose in the article a new extended diagram of the measurement system (Fig. 1a) with description of its elements.

2. ***Morphology of the utilized membrane is missing; you can use any of mentioned techniques AFM, SEM or TEM and add its analysis in your manuscript.***

The structure of the membrane is in the form of a multilayer networks of interwoven cellulose microfibers, compressed under sufficiently high pressure and with drained water. Cellulose fibers have a cross-sectional diameter of 0.1 to 0.2 µm, while their length is several µm. This structure of the network of intertwined fibers gives the membrane great flexibility and tear strength. The membrane has a layered structure, each layer consisting of cellulose fibers arranged in different directions relative to each other. The thickness of the membrane in dry state can be estimated at about 10 µm (1), while the hydration of the membrane causes its thickness to almost double to 18-20 µm (2). Hydration of the membrane makes the membrane lose its rigidity. Therefore, it is more flexible, but at the same time it is easier to place it on the skin surface without air bubbles under the dressing. The multilayer structure of the membrane with entangled fibers of bacterial cellulose makes the holes through which the solution penetrates small, of the order of 0.1 µm, with high tortuosity along the entire length of the membrane. For this reason, this membrane, as a dressing, is an excellent barrier to bacteria (3).

In the article, we supplemented the description of the membrane with its structural and transport properties, we included the SEM image of the membrane (Fig. below – in article Fig. 1b), with extended scheme of measurement system (new proposition) and based on additional literature, we extended the description of bacterial cellulose membrane applications, especially in medical applications.

A	B

Fig. 1 A – measurement system in configuration A; M_CB_ – bacterial cellulose membrane, M ‑ motor, m – magnet, mV – milivoltmeter, Com – computer. B - Cross-section of dry bacterial cellulose membrane (*Biofill*) and part of the surface of the membrane, obtained by means of a Zeiss Supra 35 with magnification 23 450.

3. ***Why only NaCl is used as electrolyte whether it will work on KCl, or any uni-uni electrolyte, how it will affect the result if uni-bi or bi-bi electrolytes were taken in account, explain your answer in conclusion.***

During our series of research, we try to use one type of solute (one electrolyte in one article), in order to obtain consistent and repeatable measurement results. In previous articles (4), (5) (6) (7) we also used KCl and other membranes (Nephrophan, UltraFlo, Bioactive Textus, … ) used in medicine. In general, the K^+^ and Na^+^ ions differ from each other in the transfer numbers for aqueous solutions and for the membrane. This leads to the fact that the observed potential differences in membrane systems and for the same initial concentration ratios on the membrane for different electrolytes are different. We observed larger potential differences at the same initial concentrations and in the same measurement systems for KCl solutions than for NaCl solutions. The differences depend on the initial concentration ratios on the membrane and are in the order of 10-40 mV. However, on the basis of the results so far, it can be concluded that the shapes of both time and concentration voltage characteristics are similar for both electrolytes. This is important for studying the time evolution and hydrodynamic stability of concentration boundary layers (CBLs). The main features of these characteristics, i.e. the time evolution of CBLs (as a result of diffusion in CBLs and permeation through the membrane), the appearance of hydrodynamic instabilities in CBLs, their main characteristics (such as intensity and duration) can be analysed by the use of both types of electrolytes ( when using Ag | AgCl electrodes, reversible to these ions) and various types of membranes. However, bacterial cellulose membranes are electro-neutral and have good transport parameters of aqueous electrolyte solutions, thanks to which the observed potential changes for selected ions are also greater, which allows for easier interpretation and analysis of the studied phenomena.

According to the reviewer's suggestion, we have outlined this problem in our conclusions.

4. ***Which bacterial membrane was utilized is not mentioned; also add extraction and preparation process of stable film.***

Bacterial cellulose membrane: *Biofill*, Fibrocel Productos Biotechnologicos Ltd. Curitiba , Brazil.

Bacterial strain producing cellulose fibers: *Acetobacter Xylinum*.

In the methodology section of the article, we have provided detailed information about the membrane used in the measurements.

5. ***XRD, FTIR is required.***

In a series of our publications the purpose of the analysis has been the phenomena occurring near the surface of the membrane in a gravitational field. Measurements with the use of various membranes (Nephrophan, UltraFlo, Textus Bioactive,…) made it possible to choose the bacterial cellulose membrane (*Biofill*) as the best one in terms of reproducibility of the obtained results and easier analysis, especially phenomena connected with hydrodynamic instabilities in CBLs caused by density gradients (concentration gradients) in CBLs directed opposite to the gravitational field acceleration. The model we used is based on the Kedem-Katchalsky equations, in which the membrane is treated as a “black box” - we are less interested in its structure, while its transport coefficients are important.The membrane material has not been structurally tested by us (XRD or FTIR) also due to the fact that there are valuable publications in which such analyses have been performed.

6. ***How does temperature affect the result?***

In our research, we focused on studying stability of the near-membrane layers in the Earth's gravitational field. We did not perform measurements at temperatures other than room temperature, 295 K. The measuring system, made of plexiglass vessels filled with solutions and with a mechanical solution mixing system, is sensitive to temperature changes. For this reason, the measurements were made at a temperature that is easy to control. As we expected, changes in the measurement temperature may affect the values of the measured potentials, but to a lesser extent, the main features of the time and concentration characteristics of the studied phenomenon, the stability of CBLs (one can expect a change in the dynamics of these phenomena and other steady states related to it). We will consider the reviewer's suggestion to extend the measurements at temperatures other than room temperature, which we will consider in future publications.

We did not extend the results to measurements at various temperatures (due to the broadness of the topic), but we consider the reviewer's remark valuable and we will take it into account in future research.

**AD. Rev. 2**

***In artificial systems, under the conditions of relationship between the density gradient and the gravitational field, give the region the reduction in concentration gradients on the membrane.***

***A divergence of the voltage concentration characteristics between the electrodes in steady states is observed, while the increase in Ch/Cl. Why? What is novelty of the present work?***

In the case of NaCl (and KCl) solutions, the solution density depends on the electrolyte concentration. This dependence can be presented (in the range of low NaCl electrolyte concentration values) as

$\rho= \rho_{o} (1+ \alpha_{NaCl} C_{NaCl}$)

where $\alpha_{NaCl}= \frac{1}{\rho_{o}} \frac{\partial\rho}{\partial C}$ , and experimentally determined equals : α_NaCl_ = 0.0414 ± 0.0003 dm^3^/mol.

As the measurements were made in the range of low NaCl concentrations, the above linear relationship between the density of the solutions and the concentration of NaCl can be assumed with a good approximation. This allows for the conclusion that the concentration characteristics of the studied phenomenon will be the same in nature and type as the density characteristics. For this reason, the conclusions drawn from the concentration characteristics (initial voltages, steady states, times of pulsation appearances, amplitudes and frequencies of the observed phenomena) will also apply to the density characteristics in the gravitational field. From the concentration characteristic of voltages in steady-states U(C), for which we observe the bifurcation of the characteristics for configurations A and B, we can assume that the initial concentration quotient at which the bifurcation begins (Ch/Cl = 50) is the quotient of the initial concentrations for which hydrodynamic instabilities appear in CBLs, and the transition is observed from the diffusive conditions of CBLs reconstruction to diffusive-convective conditions. (Ch/Cl = 50) ⇔ (ρh/ρl = 1.00000229)

In the article, we tried to clearly write about this limiting value of the concentration quotient, with the conversion of this value into the limiting value of the density quotient (according to the above equation), at which the symmetry between the configurations of the system A and B is disturbed by gravity.

In our previously published research results we focused on the case where the electrodes were immersed on both sides of the membrane. In this case, when we measured voltages, we also took into account the potential difference across the membrane and potential changes connected with both layers of CBLs at the membrane. In the current publication, for the first time, we present the results of the measurements analysed for electrodes immersed in one of the chambers with an electrolyte solution with a lower concentration. We chose a chamber with a lower electrolyte concentration due to the fact that the higher the electrolyte concentration between the electrodes, the lower voltages we observed (explanation in the article). The initial zero voltages were the starting point, and their changes over time, due to the time evolution of the CBL in the lower concentration chamber, can now be associated only with changes in one of the CBLs and not with changes of potentials through the membrane and second CBL. We believe that this measurement method should give a better picture of changes in the CBL in the chamber with a lower NaCl concentration.

In conclusion, on the basis of the results presented in the article, we can state that a more reliable approach (more effective) to the time-evolution analysis of CBL is to use a measuring system with Ag|AgCl electrodes placed in the same chamber of the system. Additionally, to observe larger potential differences between the electrodes, they should be placed in the chamber with a lower electrolyte concentration.

References

1. S. Grzegorczyn, A. Ślęzak, Kinetics of concentration boundary layers buildup in the system consisted of microbial cellulose biomembrane and electrolyte solutions, Journal of Membrane Science 304 (2007) 148-155.

2. K. Dworecki, A. Ślęzak, S. Wąsik, Temporal and spatial structure of the concentration boundary layers in membrane system, Physica A, 326 (2003) 360-369

3. M. Kucharzewski, A. Ślęzak, A. Franek, Topical treatment on non-healing venous leg ulcers by cellulose membrane, Phlebologie 32 (2003) 147-151.

4. S. Grzegorczyn, A. Ślęzak, The role of mechanical pressure difference in the generation of membrane voltage under conditions of concentration polarization, *Journal of Biological Physics* 42 (2016) 383-398.

5. A. Ślęzak, J. Jasik-Ślęzak, S. Grzegorczyn, I. Ślęzak-Prochazka, Nonlinear Effects in Osmotic Volume Flows of Electrolyte Solutions through Double-Membrane System, *Transport in Porous Media* 92 (2012) 337-356.

6. A. Ślęzak, S. Grzegorczyn, I.H. Ślęzak, A. Bryll, Study on the volume and solute flows through double-membranous polymeric dressing with silver ions, Journal of Membrane Science 285 (2006) 68-74.

7. A. Ślęzak, S. Grzegorczyn, K. Batko, W. Pilis, R. Biczak, Membrane transport in concentration polarization conditions: Evaluation of *S*-entropy production for ternary non-electrolyte solutions, *Journal of Non-Equilibrium Thermodynamics*, 45 (2020) 385-399.
